# Muscle strength, gait, and balance in 20 patients with hip osteoarthritis followed for 2 years after THA

**DOI:** 10.3109/17453671003793204

**Published:** 2010-04-06

**Authors:** Anton Rasch, Nils Dalén, Hans E Berg

**Affiliations:** ^1^Stockholm Spine Center, Löwenströmska Hospital, Upplands Väsby; ^2^Division of Orthopaedic Surgery, Department of Clinical Sciences, Karolinska Institutet, Danderyd Hospital, Stockholm; ^3^CLINTEC, Department of Orthopedic Surgery, Karolinska University Hospital Huddinge, StockholmSweden

## Abstract

**Background** Patients with hip osteoarthritis (OA) have muscular weakness, impaired balance, and limp. Deficits in the different limb muscles and their recovery courses are largely unknown, however. We hypothesized that there is persisting muscular weakness in lower limb muscles and an impaired balance and gait 2 years after THA.

**Patients and methods** 20 elderly patients with unilateral OA were assessed before, and 6 and 24 months after surgery for maximal voluntary isometric strength of hip and knee muscles and by gait analysis, postural stability, and clinical scores (HHS, SF-36, EuroQoL).

**Results** Hip muscles showed a remaining 6% weakness compared to the contralateral healthy limb 2 years after THA. Preoperatively and 6 months postoperatively, that deficit was 18% and 12%, respectively. Knee extensors fully recovered a preoperative 27% deficit after 2 years. Gait analysis demonstrated a shorter single stance phase for the OA limb compared to healthy limb preoperatively, that had already recovered at the 6-month follow-up. Balance of two-foot standing showed improvement in both sagittal and lateral sway after operation. All clinical scores improved.

**Interpretation** Muscle strength data demonstrated a slow but full recovery of muscles acting about the knee, but there was still a deficit in hip muscle strength 2 years after THA. Gait and balance recovered after the operation. To accelerate improvement in muscular strength after THA, postoperative training should probably be more intense and target hip abductors.

## Introduction

Reduced lower limb muscle strength has been shown to predict the onset of dependency in activities of daily living (ADL) in the elderly (Rantanen et al. 2002). Specifically, studies have demonstrated a relationship between muscle strength and walking capacity (Rantanen et al. 1994, Manini et al. 2007) or postural stability (Fiatarone et al. 1990). Patients with hip OA have a substantial loss of muscular strength in the affected limb compared to the healthy limb preoperatively (Arokoski et al. 2002, Suetta et al. 2004, Rasch et al. 2007), but the ability of these muscles to recover postoperatively has still not been proven (Horstmann et al. 1994, Shih et al. 1994, Trudelle-Jacksson et al. 2002).

We have demonstrated a slow morphological recovery in OA, manifest as a persistent deficit in cross-sectional area (CSA) and radiological density (RD) of hip muscles compared to the healthy limb 2 years after THA (Rasch et al. 2009). These morphological findings plus the fact that knee OA patients have shown persistent muscular deficit years after TKA (Mizner et al. 2005) led us to hypothesize that the muscular weakness of several lower limb muscles along with impaired gait and balance would be maintained 2 years after THA.

## Patients and methods

22 patients (mean age 67 (SD 7) years, mean height 168 (6) cm, mean weight 79 (16) kg, 18 females) with unilateral hip OA planned for total hip replacement were recruited consecutively between January and May 2005. They were tested on the day before surgery, and 6 months and 2 years after THA. The subjects included had no previous surgery of the lower extremity, no neurological or advanced cardiopulmonary diseases, and no lower extremity co-morbidity. 2 patients were excluded after surgery, 1 due to a peroperative femur fracture and 1 due to operation with a lateral approach. All other patients were operated with the posterior approach (Moore). At the 6-month follow-up there were 3 drop-outs (1 muscular tear just before measurements, 1 patient was abroad, and 1 patient did not want to attend) but at the 2-year follow-up 20 patients were examined. 1 patient had an early postoperative hip dislocation treated with a brace for 6 weeks, but could participate in all examinations. 5 patients were unable to perform the preoperative gait analysis and 1-foot standing, and 1 patient could not perform the 2-foot standing because of pain and the use of crutches. All 20 patients were measured for gait and postural control at the 6-month and 2-year follow-up.

We used 2 types of hip prostheses: a cementless porous-coated femur stem (Bi-metric; Biomet Inc., Warsaw, IN) (n = 8) and a cemented polished and tapered femur stem (CPT; Zimmer Inc., Warsaw, IN) (n = 12). A cemented, highly crosslinked polyethylene cup (Muller; Stryker Howmedica Inc., Rutherford, NJ) was used in all patients. All patients were preoperatively planned in order to restore anatomical offset. Postoperatively, the same regime was used for all patients who were allowed immediate weight bearing.

All patients completed 10 sessions of weekly group training after operation, and thereafter home exercises were encouraged. We used a traditional rehabilitation program using the patient's own body weight as resistance. All groups of muscles working about the hip and knee were activated. The program also included equilibrium exercises, 1-limb standing, and walking on the spot.

At the 2-year follow-up, training habits varied between individuals and ranged from no exercise to exercise several times per week. Clinical scores (SF-36, Harris hip score, and Euroqol) were collected from all patients. The subjective severity of hip pain was rated using measurements on the visual analog scale (VAS) with range 0–10. All patients provided written informed consent before participation. The experimental protocol was approved by the ethics committee of Karolinska Hospital (2003/735 and 2006/1492-32).

### Assessment of muscle strength

The test apparatus and protocol for maximal voluntary isometric force measurements have been described in detail elsewhere (Rasch et al. 2005). Briefly, the subject is first seated with the hip and knee positioned at right angles, and knee extension or flexion force is measured at the ankle via a padded brace connected to a strain gauge. After repositioning to the standing position, with the upper body supported by an inclined abdominal pad and the contralateral foot partly weight bearing onto an adjustable floor pad, hip extension, flexion abduction, and adduction force are measured via the padded brace repositioned just above the knee. The patient was instructed to contract maximally without kicking and to sustain maximal force for 3–5 sec. The mean of 2 trials at maximal effort, separated by 20 seconds of rest, was used for comparisons. Data were sampled and processed using a dedicated PC system (MuscleLab; Ergotest, Langesund, Norway). To ensure that different muscles were acting about different joints, averages were formed for knee extension/flexion (Ktot) and hip extension/flexion/abduction/adduction (Htot). Also, an average of all 6 measurements (Tot) was calculated to be able to detect small changes in force over time. Average knee force (Ktot), hip force (Htot), or total limb force (Tot) were obtained by calculating the arithmetic means of individual measurements. Reproducibility (CV%) of muscle strength measurements varied between 4% and 12% ( Rasch et al. 2005).

### Gait analysis

A flat opto-sensor walkway with 2 separate lanes—equipped with photocells in order to assess right and left foot contact times—was used. This system (IVAR Jump and Speed Analyzer, Estonia) was originally designed for measurement of contact and flight times in runners using 1 lane only; and not discriminating data from right and left feet. We have developed this method further and our custom version allows the measurement of touch-down and lift-off of both feet separately when walking on the parallel right and left foot lanes without crossing the midline. The equipment has been validated and described in detail elsewhere (Viitasalo et al. 1997, Rasch et al. 2005). Reproducibility (CV%) varied between 4.5% and 7.4%. Briefly, 1 bar containing 4 light transmitters is placed at the end of each lane. They send infrared light beams, which are individually received by four matching photocells mounted in a bar at the start of the walkway. Transmitters and photocells are placed approximately 6 mm above the flat walkway and 50 mm apart, allowing the detection of touch-down and toe-off of right and left foot across two 150-mm-wide lanes, respectively. Ground contact times less than 0.2 seconds are filtered and thus not registered, in order to block false data input because of shuffling. The above 4 detected signals are streamed to an electronic box, and later ported and stored.

Each test session comprised 3 trial runs without shoes. Starting with the right foot, a relaxed walking speed was maintained while not crossing the midline, and at least 10 steps (5 gait cycles) were measured. A typical gait cycle consists of 4 phases, including 2 single-stance phases separated by 2 double-stance phases. Step frequency (steps per second) and single and double stance duration (expressed in seconds or as a percentage of the gait cycle), were calculated. Means of each variable were derived from 5 gait cycles. The first cycle of each run was excluded since many individuals swayed during their first steps.

### Assessment of postural stability

A force plate (MuscleLab; Ergotest, Langesund, Norway) connected to the computer and dedicated software was used to analyze lateral and sagittal sway. The patients were told to stand still on the force plate with a gap of 20 cm between the feet. 6 measurements of bilateral standing with alternating open or closed eyes were first conducted, followed by 6 measurements standing on 1 foot—alternating OA and healthy limb—with eyes open. To facilitate unilateral standing in these sedentary patients, the patients were allowed to stabilize themselves with a rod in the contralateral hand. The rod was placed 10 cm in front of the lifted foot with the upper arm in contact with the trunk and the elbow in 90 degrees of flexion. Each test lasted 30 s, followed by 20 s of rest. From the 3 tests collected for each position, the mean of the 2 best measurements was used for comparisons. The sway path (movement of the center of gravity) was assessed as the standard deviation of movements in lateral and sagittal (anterior-posterior) direction, respectively. This method of quantifying postural stability has shown moderate-to-excellent reproducibility (Ekdahl et al. 1989, Goldie et al. 1989, Birmingham 2000).

### Statistics

Statistical analysis was performed using the paired t-test or ANOVA for single measures, setting the significance level at p < 0.05. For repeated measures, a 2-factor ANOVA (limb × time) was used, with a lower significance level (p < 0.03), where an error rate for multiple comparisons of simple main effects was calculated partitioning the family of the main factors and the interaction term (0.05 × 3 = 0.15) and dividing by 5 planned comparisons (Kirk 1995, p 389).

## Results

Preoperatively, all muscles except knee flexors showed a deficit of 9–27% (p < 0.03) in the OA limb compared to healthy limb (Table 1). 6 months postoperatively, that deficit was 8–16% (p < 0.03) in all muscles except hip adductors and knee flexors. 2 years after the operation, only hip abductors showed a remaining 15% (p < 0.001) deficit while knee flexors were 11% stronger in the OA limb (p = 0.02). 2 years after surgery, all muscles in OA limb had shown statistically significant improvement, except for hip adductors and knee flexors. Healthy limbs showed no statistically significant postoperative changes.

**Table 1.** Mean values of force (in Newtons (SD)) in the OA limb compared to the healthy limb in 20 patients with unilateral hip OA. The means of individual differences (Diff) between the limbs are expressed as percentages (2-factor ANOVA model)

	OA	Healthy	Diff (%)	p-value (diff)	p-value (OA)	p-value (healthy)
Knee extension						
Preop.	295 (117)	399 (92)	–27	< 0.001	0.001	0.04
6 months	329 (122)	369 (117)	–8	0.03		
2 years	367 (113)	372 (114)	–1	0.5		
Knee flexion						
Preop.	143 (44)	151 (49)	–4	0.1	0.03	0.7
6 months	147 (64)	146 (51)	1	0.9		
2 years	165 (66)	147 (47)	11	0.02		
Hip extension						
Preop.	215 (98)	266 (116)	–19	< 0.001	< 0.001	0.04
6 months	252 (110)	292 (101)	–15	0.005		
2 years	299 (114)	311 (104)	–4	0.3		
Hip flexion						
Preop.	205 (63)	270 (72)	–24	< 0.001	0.02	0.09
6 months	217 (60)	251 (66)	–10	0.004		
2 years	246 (79)	263 (67)	–7	0.07		
Hip abduction						
Preop.	140 (63)	161 (59)	–15	0.02	0.01	0.05
6 months	120 (44)	148 (46)	–16	0.002		
2 years	152 (68)	178 (73)	–15	< 0.001		
Hip adduction						
Preop.	222 (68)	249 (72)	–9	0.02	0.2	0.8
6 months	225 (66)	244 (70)	–3	0.1		
2 years	242 (75)	244 (78)	0	0.8		
Knee total						
Preop.	219 (70)	275 (63)	–21	< 0.001	< 0.001	0.09
6 months	238 (89)	258 (78)	–6	0.09		
2 years	266 (82)	259 (76)	3	0.4		
Hip total						
Preop.	195 (64)	237 (68)	–18	< 0.001	< 0.001	0.06
6 months	203 (63)	234 (63)	–12	< 0.001		
2 years	235 (75)	249 (73)	–6	< 0.002		
Total						
Preop.	203 (63)	249 (64)	–19	< 0.001	< 0.001	0.08
6 months	215 (70)	242 (66)	–10	< 0.003		
2 years	245 (75)	253 (72)	–3	0.06		
Diff: differences;						
Knee total: average knee extension/flexion;						
Hip total: average hip extension/flexion/abduction/adduction;						
Total: average of all 6 measurements;						
p-value (diff): p-value for side differences;						
p-value (OA): p-value for changes in OA limb over time;						
p-value (healthy): p-value for changes in healthy limb over time.						

Gait analysis showed a shorter single-stance phase in OA limbs than in healthy limbs preoperatively (p < 0.001) (Table 2). At the 6-month and 2-year follow-up, there were no statistically significant differences between the limbs. In the OA limbs, single-support phase increased postoperatively, but only statistically significantly when using percentage of gait cycle (p = 0.005). No statistically significant change for single support phase was observed in healthy limbs.

**Table 2.** Mean values (SD) of single support phase for OA limb and healthy limb expressed in seconds and percentage of gait cycle, and double support phase and step frequency in 16 patients with unilateral hip OA (2-factor ANOVA model)

	Single support (sec)				Single support (%)						
									Double supp		Steps/sec
	OA limb	Healthy limb	Diff	p-value	OA limb	Healthy limb	Diff	p-value	(sec)	(%)	
Preoperatively	0.55 (0.11)	0.62 (0.10)	–0.07	< 0.001	34 (4.4)	38 (2.8)	–4.1	< 0.001	0.23 (0.10)	27 (6.3)	1.3 (0.3)
6 months	0.64 (0.13)	0.63 (0.13)	+0.01	0.7	39 (3.2)	38 (4.2)	+2.0	0.4	0.21 (0.12)	23 (6.1)	1.3 (0.2)
2 years	0.60 (0.10)	0.57 (0.10)	+0.03	0.05	39 (3.5)	37 (4.1)	+2.1	0.06	0.20 (0.10)	24 (6.5)	1.3 (0.2)

Sway measurement of unilateral standing before and after operation showed no statistically significant differences between OA limbs and healthy limbs except for the 6-month follow-up of sagittal sway, where it was greater in the OA limb (p = 0.02, Table 3). Measurement of bilateral standing showed a reduced lateral and sagittal sway postoperatively compared to preoperatively, although this was only statistically significant with eyes closed (p = 0.006) (Table 4). The sagittal sway was more pronounced than the lateral sway in both OA and healthy limbs, both preoperatively and postoperatively.

**Table 3.** Mean values (mm (SD)) of sagital and lateral sway in 1-foot standing on a force plate in 16 patients with unilateral hip OA. The OA limb is compared to the healthy limb and differences between the limbs are expressed in per cent (2-factor ANOVA model)

Parameter		OA	Healthy	Diff (%)	p-value (diff)	p-value (OA)	p-value (healthy)
Sagital sway	Preop.	9.8 (4.1)	8.8 (3.2)	–10	0.3	0.7	0.4
	6 months	10.2 (3.1)	8.2 (2.7)	–20	0.02		
	2 years	9.6 (3.4)	8.4 (2.4)	–13	0.1		
Lateral sway	Preop.	7.3 (1.7)	6.5 (3.6)	–11	0.5	0.9	0.2
	6 months	7.5 (2.3)	5.5 (2.6)	–27	0.09		
	2 years	7.2 (2.1)	5.5 (2.7)	–24	0.08		
p-values: See Table 1.							

**Table 4.** Mean values (mm (SD)) of sagital and lateral sway with open or closed eyes in 2-foot standing on a force plate in 19 patients (1-factor ANOVA model)

Parameter	Preoperatively	6 months	2 years	p-value
Sagital sway				
Open eyes	4.5 (1.6)	3.9 (1.2)	3.5 (0.7)	0.04
Closed eyes	6.1 (2.6)	4.6 (1.3)	4.6 (1.3)	< 0.001
Lateral sway				
Open eyes	3.0 (1.6)	2.4 (0.9)	2.0 (0.7)	0.04
Closed eyes	4.1 (2.2)	2.8 (0.9)	2.6 (1.0)	0.006

The mean HHS, EQ-5D, and VAS all improved (p < 0.001) from 52 (34–65), 0.44 (0.03–0.69), and 5.2 (0–8) preoperatively to 86 (46–100), 0.85 (0.03–1.0), and 0.05 (0–1), respectively, at the 2-year follow-up. 2 years after surgery, SF-36 had improved (p < 0.001) for all domains except for general health (p = 0.11). Preoperative values were: physical function (PF) 29 (10–60), role physical (RP) 9 (0–100), body pain (BP) 28 (10–41), general health (GH) 65 (25–97), vitality (VT) 47 (10–85), social function (SF) 63 (25–100), role emotional (RE) 36 (0–100), and mental health (MH) 69 (28–92). 2 years postoperatively, these values were: PF 73 (20–95), RP 77 (0–100), BP 80 (31–100), GH 72 (25–100), VT 71 (13.3–100), SF 90 (25–100), RE 86 (0–100), and MH 86 (52-100).

## Discussion

This study is the first prospective study to cover more than 12 months after THA, and it also assessed muscle strength in both the hip and the knee. We found a persistent strength deficit in most OA limb muscles 6 months after THA—10% on average relative to the contralateral, healthy limb. 2 years after surgery, however, there was a fair restoration of muscle strength. Only the abductor muscle weakness would be of clinical significance. It appears that muscles of the hip and thigh do indeed have the capacity to recover after THA, given sufficient time. Our data on gait and postural stability confirm that there is a recovery process.

Minimally invasive surgery (MIS) has been suggested to improve recovery after THA. 2 studies have demonstrated a shorter hospital stay (Dorr et al. 2007) and a faster recovery in hip muscle strength, walking speed, and functional scores within the first year (Lin et al. 2007) after MIS, but none has demonstrated any long-term benefits compared to a conventional THA. The rationale for expecting improved recovery after MIS could be questioned, because muscle atrophy and weakness developed during years of OA and inactivity might not be reversed by reducing the operative trauma only. MIS data and our current results could be interpreted in such a way that there would be limited room for additional gain in the long-term recovery course, and hence less traumatic surgical techniques would have only minor effects on the final result. However, if a stable hip joint and regained ambulatory capacity can be obtained a few months earlier, particularly in elderly patients, this may reduce postoperative complications.

The surgical trauma of standard hip replacement has been suggested to obstruct muscular recovery in the first months after surgery, as a profound drop in knee extensor strength 5 weeks after standard THA and a persistent deficit (18%) in the OA limb compared to healthy limb 3 months after surgery was found (Suetta et al. 2004). No data on hip muscles were supplied by these authors, however. Similarly, knee OA patients showed pronounced knee extensor weakness 3–6 months after total knee arthroplasty, and it was even suggested that patients undergoing TKA would never recover their preoperative knee extensor force deficit in the OA limb (Mizner et al. 2005). Although we lack data taken before 6 months, in contrast full recovery of knee extensor strength and mass has been found by us 2 years after standard THA in hip OA patients (Rasch et al. 2009).

Muscles acting about the hip appear to be slower to recover than knee extensors after THA, suggesting that there may be local adaptation of muscles about the OA-affected joint. CT showed atrophied hip muscles but not atrophied thigh or calf muscles 2 years postoperatively (Rasch et al. 2009), and our current force data indicate that about two-thirds of the force deficit in knee extension but only one-third of the deficit in total hip muscle force had recovered at 6 months, and also that reduced hip muscle force was still detectable 2 years after THA (Table1). Similarly, Horstmann et al. (1994) found that abductor weakness had not been restored to the preoperative level 6 months after THA. Traumatization or denervation of the abductors or other hip muscles at surgery has been suggested to explain a delayed recovery or permanent loss of force capacity of those muscles. In our view, however, the chronic alteration in hip muscle tissue preoperatively (Rasch et al. 2007), suggesting that fat infiltration persisted 2 years after THA (Rasch et al. 2009), seems a more likely explanation for local differences in recovery rates. The mechanism of these tissue changes could be a long-term redistribution of weight-bearing load from muscles of the painful hip to the knee and calf muscles. We have not found any comparisons between different limb muscles in knee OA before or after TKA.

Unexpectedly, the ambulatory function was largely recovered already 6 months after surgery. Previous studies have shown that patients with hip osteoarthritis have a reduced single-stance phase of the OA limb (Olsson et al. 1986). This gait asymmetry was ascribed to pain and a reduced ability of the affected hip joint to sustain load (Kyriazis and Rigas 2002). Our preoperative data confirm the results of these studies and revealed a shorter single-stance phase in the OA limb than in the healthy limb. This limp had already recovered 6 months after surgery when the patients had received a pain-free THA, but still showed muscular weakness and atrophy. This might indicate that joint pain is a more important factor than hip abductor strength for inducing limp, as suggested by Horstmann et al. (1994).

Postural sway on quiet bilateral standing was reduced after THA as previously shown (Wykman and Goldie 1989), and the natural interpretation would be that an impaired postural stability due to OA became improved after THA. In order to evaluate limbs separately, and the role of muscles for postural stability, we assessed sway on unilateral standing with partial support of the contralateral arm. Unexpectedly, we could not find any differences between the OA limb and the healthy limb, and there was no improvement after THA. The authors of another published unilateral sway test study similarly concluded that there was no difference between the OA limb and the healthy limb (Arokoski et al. 2006). Despite the speculations about disturbed proprioceptive pathways and muscular feedback about OA joints and their removal at THA surgery, it does not appear to be proven yet that there is a clinically important impairment of postural stability due to hip OA before and after THA.

Preoperatively, our patient group scored similarly in SF-36 to patients in previous studies of hip OA (Ostendorf et al. 2004) and Harris hip score and EuroQoL were similar to data collected on patients planned for THA that have been reported to the Swedish hip registry (www.jru.orthop.gu.se). Thus, our patients appear to be representative of the typical hip OA patient who is considered for THA, and self-reported function and quality of life as well as measured index of muscle mass and function are indeed lowered. Improvements in self-reported scores have been strongly associated with improved pain rather than actual ability to perform (Stratford and Kennedy 2006). We can confirm this by demonstrating a persistent muscular weakness in the hip but a full recovery of pain on the VAS scale and a full recovery of health scores, SF-36 and EuroQoL, as compared to a normal age-matched population (Ostendorf et al. 2004, Sullivan et al. 1995). Self-reported scores are probably incapable of picking up moderate deficits in muscular strength.

In summary, within two years of standard THA, muscular weakness and function were largely recovered but the substantial deficit shown during the first 6 months may merit intervention. Less invasive surgery would be one way to speed recovery after hip replacement. Alternatively, because strength training has proven to be safe and effective in the early phase after THA (Suetta et al. 2004), supervised intense rehabilitation training programs targeting hip abductors also may be of value after standard THA.
